# Mitochondria in cone photoreceptors act as microlenses to enhance photon delivery and confer directional sensitivity to light

**DOI:** 10.1126/sciadv.abn2070

**Published:** 2022-03-02

**Authors:** John M. Ball, Shan Chen, Wei Li

**Affiliations:** Retinal Neurophysiology Section, National Eye Institute, NIH, Bethesda, MD 20892, USA.

## Abstract

Mammalian photoreceptors aggregate numerous mitochondria, organelles chiefly for energy production, in the ellipsoid region immediately adjacent to the light-sensitive outer segment to support the high metabolic demands of phototransduction. However, these complex, lipid-rich organelles are also poised to affect light passage into the outer segment. Here, we show, via live imaging and simulations, that despite this risk of light scattering or absorption, these tightly packed mitochondria “focus” light for entry into the outer segment and that mitochondrial remodeling affects such light concentration. This “microlens”-like feature of cone mitochondria delivers light with an angular dependence akin to the Stiles-Crawford effect (SCE), providing a simple explanation for this essential visual phenomenon that improves resolution. This new insight into the optical role of mitochondria is relevant for the interpretation of clinical ophthalmological imaging, lending support for the use of SCE as an early diagnostic tool in retinal disease.

## Teaser

In addition to delivering energy, cone photoreceptor mitochondria in the retina moonlight as microlenses to deliver light.

## INTRODUCTION

**Fig. 1.**
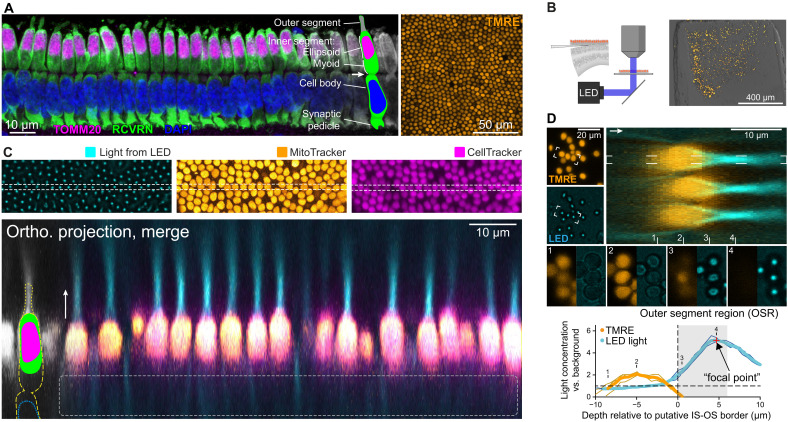
Isolated cone photoreceptor ellipsoids focus incident light. (**A**) Cone mitochondria in dorsal GS retina. Left: Cone photoreceptor anatomy in an immunolabeled vertical section. The arrow indicates the junction between the IS and cell body. Right: Top-down flat-mount view of TMRE-labeled mitochondria in live photoreceptors. RCVRN, recoverin. (**B**) Horizontal sectioning and imaging of light transmission through agarose-embedded retinas. (**C**) Top-down view of light concentration in a 3D *Z*-stack by photoreceptors in retinas sectioned as in (B). MitoTracker localizes to mitochondria; CellTracker is sequestered within the cytoplasm following internalization. In the orthogonal vertical projection through the image stack, note the absence of CellTracker labeling where the cone myoid region would ordinarily be expected [dashed line; compare to (A)]. The arrow indicates the direction of light transmission. (**D**) Quantification of light concentration factors in three exemplary cones. Flat-mount TMRE view is a maximum projection; the LED image is a single plane near the peak concentration intensity. Orthogonal projection comes from the bracketed cones. Panels (1) to (4) depict features of light concentration at the depths indicated. Graph shows the relative light intensity (concentration factor) for the three cones (individual and average) as a function of depth from the distal IS tip in the 1.5-μm-diameter OS region (OSR; top cone, dashed line; see Materials and Methods). TMRE signal is arbitrarily scaled for comparison.

Mitochondria are well known as essential organelles for energy production in eukaryotic cells. Although diverse in size, shape, number, and location in different cells and tissues, for most mammalian cells, mitochondria form a reticular network surrounding the nucleus (*1*). However, photoreceptors of the retina, especially cones, have an abundance of tightly packed mitochondria, which, in many species including primates and humans, are arranged into an elongated bundle (*2*, *3*). This bundle occupies the ellipsoid, the distal portion of the cone inner segment (IS), immediately proximal to the light-sensitive outer segment (OS; Fig. 1A). Generally, it is assumed that such a high density of mitochondria at this unusual location is optimally situated to supply ample adenosine triphosphate (ATP) to support phototransduction in the cone OS (*4*, *5*); however, increasing evidence suggests that photoreceptors rely more on glycolysis than mitochondrial oxidative phosphorylation for their energy needs (*6*).

An alternative explanation for this precise arrangement of cone mitochondria may lie in their role in shaping the path of light as it passes toward the OS. The vertebrate retina has an inverted structure with many neural layers through which light must pass before successful detection by the photoreceptor OS. Thus, there is substantial evolutionary pressure upon the retina to facilitate light delivery to the OS for detection. For instance, in nocturnal mammals, the compact arrangement of chromatin in rod photoreceptor nuclei is believed to minimize light scatter (*7*). In addition, Müller glia, support cells that axially span the retina from its inner surface to the base of the photoreceptor IS, have been shown to have optical fiber–like light guidance properties (*8*–*10*). However, the photoreceptor IS constitutes the last structure that light must pass through before reaching the OS. There, the shape of photoreceptors and their elevated average refractive index have been recognized for their similarity to the design of miniature dielectric antennas (*11*), although the subcellular components vary: In many birds and reptiles (as well as some marsupials), large (2 to 5 μm) oil droplets can be found in the distal cone IS, immediately proximal to the OS (*12*). Owing to their high index of refraction, oil droplets are expected to serve an optical role, concentrating light for detection in the OS (*13*–*16*). In mammals, such an arrangement of mitochondria is likely to have relevant optical features, owing to the high refractive index of lipid membrane (*2*, *17*–*19*), yet their complex internal geometry may instead result in detrimental light scatter. This question is particularly relevant for vision in the human fovea: Despite the displacement of scattering elements from the light path that is its hallmark (*20*), cone mitochondria remain the final obstacle to light. To date, a conclusive investigation of the optical consequences of cone mitochondria has yet to be presented, largely because of the complexity of isolating the optical nature of the cone ellipsoid from that of the remainder of the retina.

Here, we directly demonstrate the optical properties of cone mitochondria by taking advantage of distinct biological features of the thirteen-lined ground squirrel (GS), a hibernator featuring a cone-dominated retina. These properties enabled us to establish a unique experimental preparation consisting of an isolated layer of cone ellipsoids containing live mitochondria. Modification of the transverse illumination path of a confocal microscope allowed us to specifically observe the optical properties of cone mitochondria, and electromagnetic finite-difference time-domain (FDTD) modeling using high-resolution mitochondrial reconstructions corroborated and extended these findings. Here, we reveal that, far from being detrimental to light detection, cone mitochondria alone are sufficient to focus incident light to a thin pencil of light that is appropriate for detection by the cone OS. Furthermore, we demonstrate both experimentally and computationally that cone mitochondria–dependent delivery of light to the OS features an angular dependence matching that of the Stiles-Crawford effect (SCE) (*21*), a retinal feature that increases visual resolution. We find that this directionality depends directly on the effective mitochondrial refractive index, which can change depending on respiration state (*22*) and protein concentration (*23*). Together, these findings not only demonstrate that cone mitochondria have important optical properties but also indicate that noninvasive retinal imaging modalities such as optical coherence tomography (OCT) (*24*) and adaptive optics (*25*), which have revolutionized the diagnosis of retinal disease, may be poised to identify mitochondria-specific deficits given a thorough understanding of the precise details of photoreceptor optics. These new insights into the optical role of cone mitochondria therefore have imperative clinical implications.

## RESULTS

### Intact, isolated cone ellipsoids focus light

To investigate the optics of cone mitochondria, we took advantage of two unique and useful properties of the GS (*26*). First, in contrast to most rod-dominated animal models used in vision research, the GS has a retina in which more than 85% of photoreceptors are cones (*27*) and that contains a single layer of photoreceptor cell bodies in its dorsal extent (Fig. 1A). Second, as an obligate hibernator, the GS endures near-freezing body temperatures for several months during the winter with remarkable hypothermia- and hypoxia-tolerant cellular specializations that render ex vivo tissue stable for up to several days (*28*–*30*). Here, these specializations permit us to devise a preparation for measuring the optical properties of live, mitochondria-packed cone ellipsoids. This was done by horizontally sectioning agarose-embedded GS retinal explants via vibratome to isolate the cone layer (Fig. 1B). The resulting samples featured patches of photoreceptors that often retained their native pseudo-hexagonal packing; their survival was verified by live labeling with tetramethylrhodamine ethyl ester (TMRE), a cationic membrane-permeable fluorophore that localizes to mitochondria because of their strongly negative inner membrane potential, providing both a clear indication of mitochondrial viability and a convenient marker for localization (Fig. 1, A and B). Serendipitously, these preparations frequently featured cones with cell bodies removed but nevertheless retained intact ellipsoids, as assessed by post hoc immunolabeling including nuclear staining with 4′,6-diamidino-2-phenylindole (DAPI; fig. S1) and during live imaging by Hoechst staining of cell nuclei and the use of CellTracker (Fig. 1C). These preparations were ideal for observing the optical properties of live cone mitochondria. We speculate that the physical stress of sectioning may have caused photoreceptors to preferentially break at the cell body–IS junction (the narrowest part of the cell; arrow in Fig. 1A), after which the plasma membrane sealed, leaving behind only the ellipsoid and its mitochondrial contents [Fig. 1D (cartoon) and fig. S1A]. This notion is supported by observations that this region is fragile and may be the source of detachment in certain retinal diseases (*31*, *32*). In addition, this preparation involved the removal of the retinal pigment epithelium, which typically causes the detachment of the delicate OSs that it tightly ensheathes. The presence versus absence of OSs did not impart any evident systematic differences upon the optical data presented below. Thus, although OSs likewise have optical properties that will influence photon absorption, the preparation presented here allowed us to independently observe and focus upon the optical role of the cone ellipsoid in the delivery of light to the OS.

**Fig. 2.**
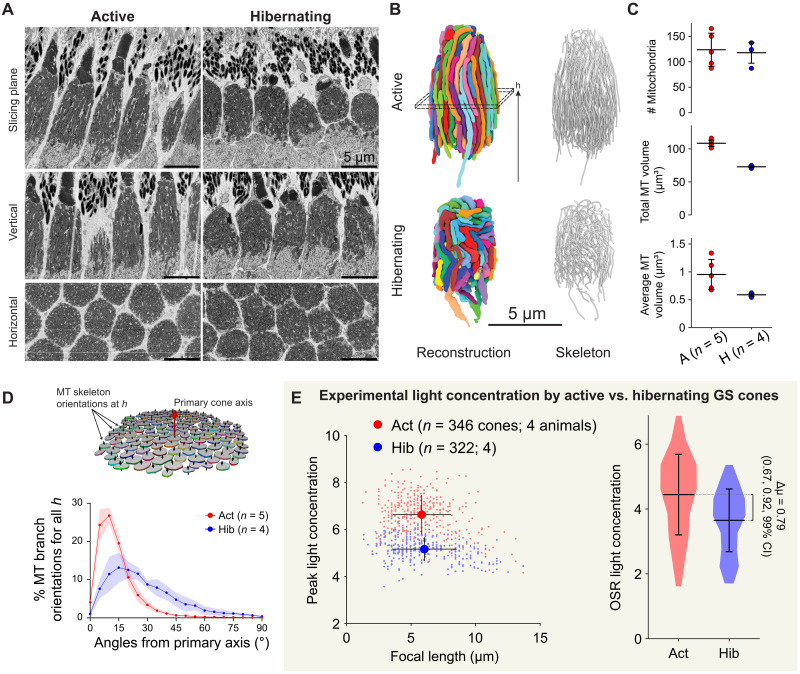
Cone mitochondrial structural changes correlate with hibernation-induced decreases in light gathering. (**A**) Serial block-face electron microscopy (SBEM) images of GS cone mitochondria. Vertical and horizontal images are software projections. (**B**) Example reconstructions (segmented 3D models and skeletonizations) of all mitochondria from sample cones. (**C**) Morphological quantification of reconstructed mitochondria. Data shown as means ± SD. Statistical comparison not performed here (see Materials and Methods). MT, mitochondria. (**D**) Quantification of mitochondrial alignment from skeletons. The diagram depicts the scheme for measuring mitochondria branch orientation at an example cone height *h* [see (B)]. The graph shows histograms (means ± SD; shaded regions) of mitochondrial deviation angles throughout reconstructed cones. (**E**) Experimental light concentration comparison for active versus hibernating GS cones (see Fig. 1). The scatterplot shows cone focal points and means ± SD for each condition. Violin plot shows the distributions of average light concentration in the OSR and the 99% confidence interval (CI) of the difference in means (see Materials and Methods for a detailed explanation of statistics). Act, active; Hib, hibernating.

To simultaneously observe TMRE fluorescence and the influence of mitochondria on passing light, we replaced the condenser of a laser scanning confocal microscope with a dichroic mirror to direct the light from a blue-filtered white collimated light-emitting diode (LED; 490-nm cutoff) toward the sample (Fig. 1B). Thus, we were able to acquire confocally sectioned three-dimensional (3D) image stacks of the light distribution passing through cones and their mitochondrial TMRE fluorescence for spatial registration (Fig. 1, B to D). Notably, LED light passing through cones was concentrated to pencils of light that, at their brightest points, were many times brighter than the background light intensity (Fig. 1D and movie S1). These pencils originated near the proximal end of ellipsoids as weak “halos” of light that coalesced to bright spots (termed “focal points” here) located a few micrometers beyond the distal end of the IS and having a half-width of ∼1 μm (Fig. 1D), very similar to the location and diameter of the GS cone OS (see Fig. 2A). Such placement of a concentrated beam of light would greatly enhance entry of photons into the OS (facilitating IS/OS coupling) and, thus, their detection. Cones with shorter focal lengths would be better suited to couple this intense beam of light into the proximal end of the OS, which may then guide this light along its length, maximizing detection. Notably, in rare samples, sectioning yielded well-preserved patches of isolated intact cones including full ISs and cell bodies. Cones in these patches also focused light well and typically at short focal lengths near the shortest of the range seen in isolated cone ellipsoids (fig. S2), supporting the prediction that these focal points occur at shorter distances in vivo.

### Optical consequences of mitochondrial remodeling

To further probe the relationship between mitochondrial structure and this concentration of light, we took advantage of the hibernating GS, whose cone mitochondria have been reported to undergo structural changes that include a reduction in total number and/or volume (*33*–*35*). Such structural remodeling may impart measurable optical consequences to the cone ellipsoid. To collect more precise details about such remodeling, we first performed mitochondrial reconstructions from serial block-face electron microscopy (SBEM) in the dorsal region of the retina from an active and a hibernating GS (Fig. 2, A and B). Cone mitochondria in the hibernating dataset were as numerous as those in active squirrel but were individually smaller, resulting in a lower total mitochondrial volume in hibernating cones [30% lower; see Fig. 2 (B and C)]. Notably, however, we also found that whereas mitochondria from active squirrel appeared elongated and well organized, those in hibernating squirrel were distorted and markedly less well aligned (Fig. 2B). A detailed analysis of mitochondrial skeletons demonstrates exceptional alignment among cone mitochondria from active GS: Approximately 75% of mitochondria branches reconstructed from active cones deviated by less than 15° from their aggregate orientation (see Materials and Methods and fig. S3 for details) compared to only 30% in hibernating cones, indicating considerable relative disorganization in the latter (Fig. 2D and fig. S3).

In our data demonstrating light focusing by cone ellipsoids, we also observed differences when we separated results from active versus hibernating squirrels. Focusing in hibernating samples was weaker than in those from active samples (Fig. 2E). Specifically, while focal lengths were similar, although variable, in both active and hibernating samples (active, 5.8 ± 2.2 μm; hibernating, 6.0 ± 2.5 μm), peak intensity was higher in active (6.6 ± 0.8–fold brighter than background) than in hibernating samples (5.2 ± 0.5–fold). To estimate the overall effect of these differences on the light theoretically available to the cone OS, we integrated the total LED light intensity within a cylinder centered upon the TMRE signal and measuring 6 μm long and 1.5 μm in diameter, approximating the anatomical OS region (OSR; Fig. 2E). Average light concentration in the OSR was ∼22% higher in active samples (4.44 ± 1.25–fold versus 3.65 ± 1.0–fold increase). Bootstrap statistics were used to determine that the 99% confidence interval for this difference did not include zero (range [0.67, 0.92]; see Materials and Methods), indicating a statistically significant increase in focusing power in cones from nonhibernating samples. These differences support the hypothesis that the elongated, parallel organization of cone mitochondria enhances the concentration of light for detection in the OS.

**Fig. 3.**
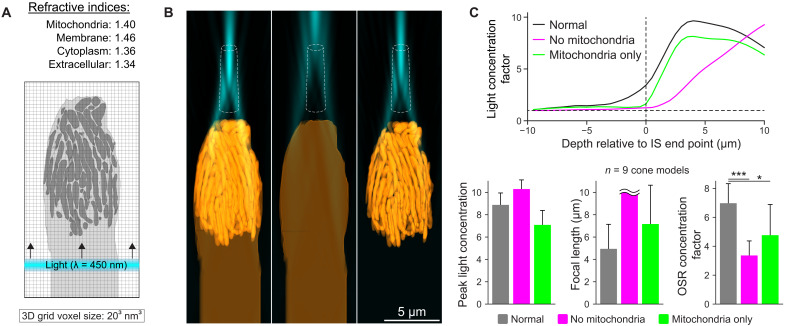
Electromagnetic simulations of light concentration by GS cone mitochondria. (**A**) 2D schematic of the 3D FDTD simulation framework. (**B**) Example light concentration by a reconstructed cone from active GS. From left to right: Intact, without mitochondria, and mitochondria only. The dashed shape depicts approximate OSR. (**C**) Quantification of simulated light focusing. The top graph depicts light concentration factor profiles for simulations shown in (B). Bar graphs show aggregate measures across all reconstructions (*n* = 9). Values are means ± SD; wavy lines indicate apparent focal lengths that fell beyond the upper boundary of the simulation volume (>10 μm). *P < 0.05, ***P < 0.001.

In these experiments, optical differences between active and hibernating squirrels may result from a mixture of factors, such as differences in gross morphology, protein concentration (*36*, *37*), or even altered cristae structure (*22*). To distinguish among these possibilities, we turned to computer modeling of light passage through high-resolution reconstructions of cone ellipsoids using electromagnetic FDTD simulations. We converted mitochondrial reconstructions into 3D grids containing the appropriate refractive indices (Fig. 3A and fig. S4) for incorporation into code libraries from MEEP (the MIT Electromagnetic Equation Propagation FDTD simulator). In short, FDTD simulations divide a dielectric structure (in this case, an optical representation of the cone) into a grid of coordinates within which spatially varying electrical and magnetic fields are iteratively calculated using Maxwell’s equations (*38*). Light passage was simulated using a continuous-wave, linearly polarized current source (450-nm wavelength), which was placed at the proximal end of the cone, thus simulating the entrance of light into the cone IS (Fig. 3A). By marching the simulator through time and allowing the resulting electromagnetic waves to propagate and settle, we produced a theoretical 3D view of the passage of blue light through these cones that could be quantified with the same techniques used for our confocal imaging data (Figs. 1 and 2).

Qualitatively, simulated light focusing by cones matched what we observed experimentally, in which the electromagnetic fields were focused to a pencil of light coinciding with the approximate location of the cone OS (Fig. 3B). To assess specifically the relative contributions of mitochondria to their light concentration capabilities, we repeated these simulations under conditions where mitochondria were removed or the cell membrane and cytoplasm were instead removed, leaving behind only mitochondria. Cones devoid of mitochondria were capable of gathering light; however, in these simulations, the peak intensity was located at considerably longer focal lengths, where most of the electromagnetic energy would fail to efficiently couple with the OS (Fig. 3, B and C). In contrast, simulations with only mitochondria exhibited light concentration similar to that of intact cones (Fig. 3C), suggesting that mitochondria play a major optical role, without which light coupling between the IS and OS would be severely compromised.

### Mitochondrial organization is critical for effective light concentration

**Fig. 4.**
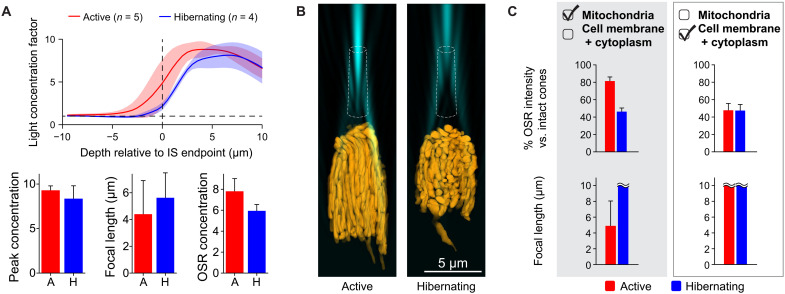
Enhanced light gathering by mitochondria in simulations of active compared to hibernating GS cones. (**A**) Quantification of light concentration in simulations of active versus hibernating GS cones. Profile shows the increase in light versus baseline intensity within a 1.5-μm-diameter range of the central cone axis as a function of depth. The shaded regions indicate the SDs from the mean across the indicated condition. Bar graphs compare measures of focusing between active (*n* = 5) and hibernating (*n* = 4) GS cone simulations. (**B**) Comparison of example simulations concerning isolated mitochondria with cell membrane and cytoplasm removed. (**C**) Quantification of light concentration measures for simulations with only mitochondria versus those with cell membrane and cytoplasm but devoid of mitochondria. Wavy lines indicate focal lengths for simulations whose apparent focal points lay beyond the simulation bounds (i.e., >10 μm).

We then asked whether the orderly arrangement of mitochondria seen in the cone ellipsoid is beneficial for light concentration. To this end, we compared light focusing in FDTD simulations of reconstructed cones from active versus hibernating samples. Corroborating our experimental imaging results, simulations of hibernating samples demonstrated weaker light concentration in the OSR (5.8 ± 0.6–fold versus 7.8 ± 1.2–fold for active GS cones; Fig. 4A). This difference resulted from both lower peak intensities and longer focal distances, slightly differing from our experimental results, which largely show a reduction in peak intensity for hibernating samples (Fig. 2E). This discrepancy likely results from other changes that may accompany the structural remodeling seen in hibernating squirrel cones, such as differences in protein concentration (*39*) or inner matrix volume (*22*), thus altering the effective mitochondrial refractive index, which we found in simulations, will, in turn, have effects on focal lengths and peak intensities (fig. S5).

Notably, in simulations containing only mitochondria, cones from nonhibernating squirrels focused light much more strongly than did their hibernating counterparts (Fig. 4B), concentrating light into the OSR 80% as effectively as for intact cone simulations compared to only 50% for simulations of isolated mitochondria from hibernating squirrel cones (Fig. 4C). Emphasizing the specific importance of the structure of cone mitochondria for optics, simulations of active or hibernating cones devoid of mitochondria were equally ineffective at concentrating light (<50% as strongly as in simulations of intact cones; Fig. 4C). These results indicate that well-aligned mitochondria bring a substantial advantage to such light concentration. Furthermore, separate simulations indicated that this elongated organization was superior for light concentration compared to volume-matched configurations consisting of roughly spherical mitochondria (fig. S6). A single “megamitochondrion” (fig. S6) proved to be a highly effective configuration for light focusing; this alternative configuration mimics the giant mitochondria seen in some cones of the tree shrew (*40*) and zebrafish (*41*, *42*) and is also similar to the “ellipsosomes” present in other species (*43*).

### Mitochondrial light focusing displays SCE-like directionality

**Fig. 5.**
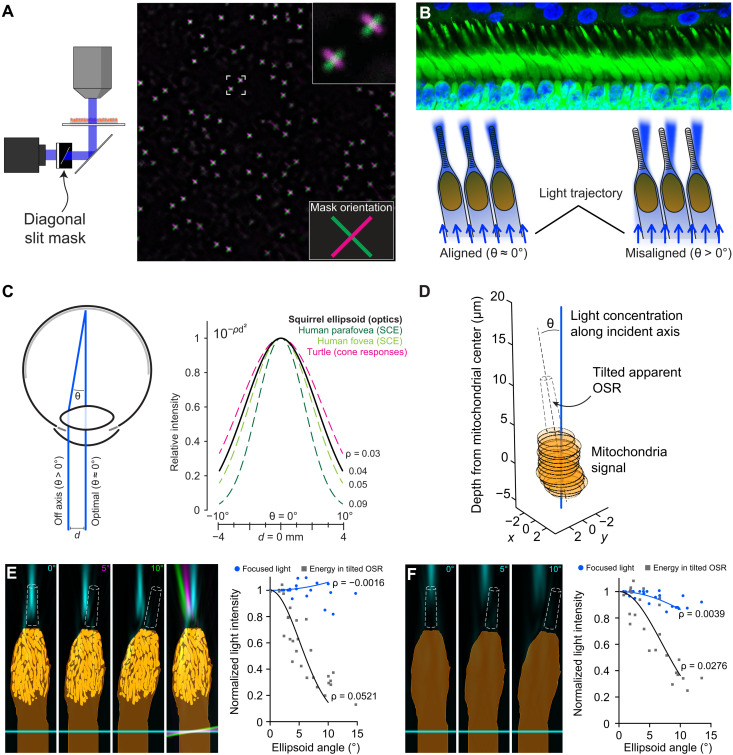
Simulated Stiles-Crawford–like directionality of light concentration by GS cone mitochondria. (**A**) Reproduction of an inverted image plane (slit mask) at the focal points of cone ellipsoids. The image is the overlaid result of two separate acquisitions with the mask reversed before the second acquisition. (**B**) Conceptual illustration of angle-dependent concentration of light upon the cone OS. (**C**) Diagram of the SCE and directionality. Angular dependence of light focusing shown here in GS is overlaid upon directionality tuning curves based on measurements in human SCE (*45*, *67*, *68*) and turtle cone light responses (*15*). (**D**) Schematic of the directionality calculation for cone light concentration in 3D space (see Materials and Methods). (**E**) Directionality of simulated light concentration in the OSR by GS cones. Left: Example simulations with models tilted by the indicated angles. Overlay shows the simulations rotated in register with one another in different colors for comparison. Graph shows the directionality fit analysis [compare to (C)] across simulations for all cone models (three angles per cone model). (**F**) Reduced directionality of simulated cone light concentration without mitochondria.

The data presented so far indicate that tightly packed, elongated mitochondria appear to maximize light delivery to the OS, likely by reducing the number of refractive interfaces encountered by photons. This strategy resembles that used by species bearing oil droplets, which act as microlenses with a homogeneous elevated refractive index (*12*, *13*); similarly, apposition-style compound eyes use such a strategy to provide individual lenses to individual light-receptive elements in noninverted retinas (*44*). Emphasizing the lens-like nature of the mitochondria-filled cone ellipsoid, we observed that isolated ellipsoids indeed reproduced an inverted image (generated by placing a mask with a slit in front of the LED) near their focal point (Fig. 5A), as has been shown for isolated avian oil droplets (*13*). Thus, each mammalian cone photoreceptor may effectively have its own mitochondria-derived microlens.

Light that is incident upon a lens at small angles that deviate from its primary optical axis will nonetheless be focused along that axis of incidence. In the situation in mammalian photoreceptors described above, this observation will bear significance for the detection of off-axis light, which, in psychophysical experiments, is perceived as having decreased brightness with increasing angles, a classic visual phenomenon known as the SCE (*21*, *45*). The SCE, the origins of which are commonly attributed to the waveguiding properties of photoreceptors (*45*, *46*), is believed to improve visual resolution and reduce veiling background (see Fig. 5C) (*21*). In well-preserved retinal sections, alignment of IS and OS is typical [Fig. 5B; see also (*47*–*49*)], and recently, it has been demonstrated that a light-dependent developmental process ensures such alignment in newborn mice (*50*). Thus, according to our optical model of mitochondria, light arriving along this axis of alignment will be maximally concentrated onto OSs; however, light entering instead at a small angle from this axis will partially miss the OS (Fig. 5B). While the relatively high refractive index of mitochondria has been noted (*2*, *51*–*53*), their role in SCE remains speculative, ranging from confounding it because of light scattering (*46*, *51*) to instead enhancing it (*2*). Here, we hypothesize that light focusing by cone mitochondria could produce SCE-like directionality, and the amount of light failing to enter OSs because of misalignment would be affected by the extent of this focusing.

To test this hypothesis, we first returned to FDTD simulations in which cone ellipsoids were tilted by angles up to 10° and measured the energy density of light available to the now-tilted OSs (Fig. 5D). By comparing this value to that found in nontilted simulations, we calculated the directionality parameter ρ, which originates from a fit to psychophysical SCE direction sensitivity data and describes the steepness of the SCE curve. Values of ρ range from ∼0.05 to 0.09 mm^−2^ for human retina [Fig. 5C; see also (*21*, *54*, *55*)], while smaller values can indicate pathological disruption of photoreceptors (*56*). Confirming our initial hypothesis, tilting at such small angles had little effect on the amplitude of on-axis light concentration; however, the loss of light available to the tilted OS agreed quantitatively with the SCE, yielding an average ρ of 0.052 across all cone models (Fig. 5E). We theorized that this measure would be sensitive to mitochondrial focal length; by repeating these simulations with mitochondria removed (which produces longer focal lengths; see Fig. 3C), we observed that the loss of light at the OSR due to tilting was diminished, effectively impairing direction sensitivity (ρ = 0.028; Fig. 5F). Thus, these simulations support the theory that the optical properties of mitochondria can specifically affect photoreceptor directional sensitivity.

**Fig. 6.**
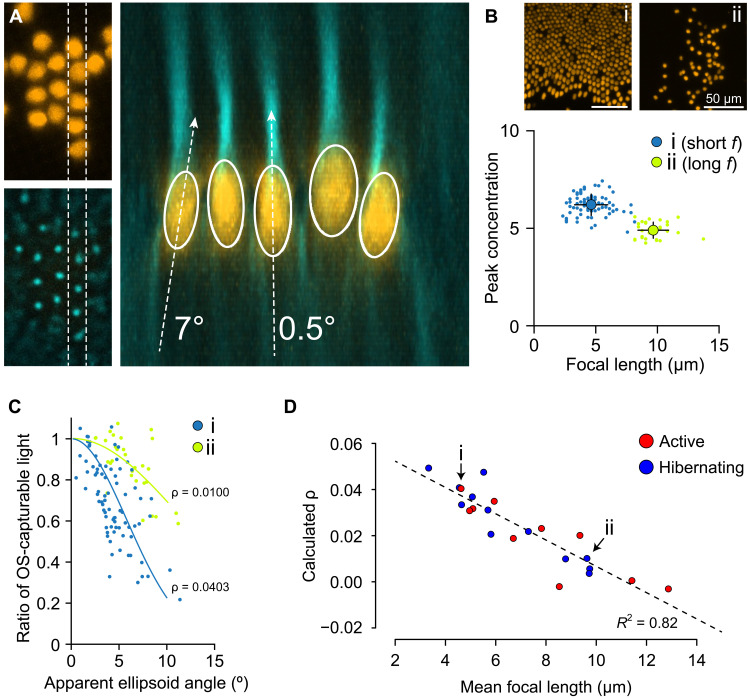
Experimental demonstration of SCE-like angular dependence of light concentration by isolated ellipsoids. (**A**) Concentration by cone mitochondria along the axis of light incidence. Annotations are simplified angle measurements in the 2D plane used here only for illustration, whereas angle measurements shown in (C) were performed in 3D space. (**B**) Example images with cones featuring short average focal lengths (i; “short *f*”) versus long average focal lengths (ii; “long *f*”). (**C**) Directionality measures for the cones for the example images shown in (C). Note the stronger directionality demonstrated by cones in the short *f* sample. (**D**) A strong correlation between mean focal length and apparent directionality (calculated ρ) across GS samples.

We then corroborated and extended these findings using experimental imaging of mitochondria focusing: Because of sample preparation and handling, photoreceptor ellipsoids displayed slight tilts of up to 15° (Fig. 6A). While the light focused by tilted photoreceptors appeared to be only minimally disturbed by misalignment, the loss of light in the estimated OSR for these cells showed a similar directional dependence to that calculated from simulations (Fig. 5). Moreover, as with simulations, samples with shorter focal lengths (likely reflecting healthier, more intact conditions; Fig. 5B) yielded larger ρ values, which were close to our simulation value and within the range of those reported for human SCE (Fig. 5C). This relationship between focal length and ρ was linear and strongly correlated (*R*^2^ = 0.82; Fig. 6D), and this correlation was apparently independent of hibernation state of the animal, supporting our theory that focal length will contribute more strongly than the amplitude of light concentration toward overall directional sensitivity. Notably, in separate simulations, we found that variations in the effective mitochondrial refractive indices or the addition of cristae-like internal structures to mitochondria had strong effects on the effective focal length (fig. S5), indicating that psychophysical directionality may be useful as a diagnostic indication of altered mitochondrial physiology (*22*).

## DISCUSSION

In summary, through live imaging and simulation, we demonstrate a clear optical role for cone mitochondria that extends their traditional role as solely serving the energetic needs of phototransduction. Using mitochondria for light concentration is advantageous over the oil droplets used by birds and reptiles because it avoids light absorption by the pigments present in the latter (*57*). This interpretation of cone optics favors focusing by mitochondria over the tapering of waveguided modes as the chief mechanism for mode matching for efficient coupling of the IS and OS (*58*, *59*). These observations will be relevant for the interpretation of images obtained using clinical ophthalmological diagnostic tools (*3*, *60*). For example, although it remains controversial regarding the structural correlation of the most prominent OCT signal band around the ellipsoid zone or IS/OS junction (*60*, *61*), our findings suggest that pathological changes in cone mitochondria may be read out as alterations in this signal band, which will be instrumental for diagnosis of human cone dysfunctions.

This demonstration of a likely role for mitochondria in the SCE-like angular dependence of light delivery to the OS provides necessary conceptual fine-tuning of the optical properties of photoreceptors. Although it has generally been assumed that long ISs with high refractive indices cause internal refraction of off-axis light within a critical angle (*21*, *45*, *46*), the model of photoreceptors as dielectric antennas has improved the agreement between theory and experiment (*11*, *51*, *59*). While demonstration of the SCE or its optical hallmarks in animal models is rare, single cone light responses in well-preserved turtle eye cups have been shown to demonstrate such directional sensitivity (see Fig. 5C) (*15*). It is tempting to speculate that focusing by oil droplets may likewise be responsible for this direction-dependent light delivery to the OS; however, comparable simulations of the oil droplet–bearing cones in chicken exhibit a much broader directional tuning curve [with an equivalent ρ < 0.005; see (*62*)]. Thus, this question requires further study with respect to the optics of oil droplets. Nonetheless, squirrel-like cone morphology is common among vertebrates, appearing even in lampreys (*63*). Altogether, direction sensitivity in nonprimate species that lack oil droplets may require the mitochondria-based optical phenomenon demonstrated here.

Regarding human retina, we propose that waveguiding by photoreceptors likely plays a strong role in central fovea (foveola), where cones have more sparsely packed mitochondria than those located even immediately outside that area (*2*, *64*, *65*). Foveolar cones may benefit from their long and narrow ISs, as our simulations indicate that simply lengthening the IS of the squirrel cone shortens its effective focal length [fig. S7; see also (*66*)]. However, SCE is weaker for foveolar cones than those outside the central fovea [(*67*, *68*); see also Fig. 5C and (*69*, *70*)], which are shorter, have larger diameters, and feature prominent mitochondria bundles resembling those in GS (*2*, *21*, *65*). There, mitochondria likely play a major role in generating SCE-like directionality of light delivery.

This sharpening of directional tuning by the presence of mitochondria, especially given the strong correlations shown here between directionality and focal length (Fig. 6D) and between focusing and mitochondrial refractive index (fig. S5), also lends support to the use of SCE as a noninvasive measurement for the diagnosis of retinal degenerations, many of which entail dysfunctions that may impart either structural or biochemical changes to mitochondria, both of which will have optical consequences (*56*, *71*). Further studies are warranted to explore structural and functional changes in cone mitochondria and their manifestations in detectable optic features.

## MATERIALS AND METHODS

### Experimental model and subject details

#### Animal care and use

Female GSs (*Ictidomys tridecemlineatus*), ranging from 7 to 15 months old (100 to 300 g) at the time that experiments were conducted were used for this study. Females were used because of their relative abundance in these litters, which were purchased from a colony managed at the University of Wisconsin—Oshkosh animal facility (*72*).

All animal procedures were preapproved by the Institutional Animal Care and Use Committee of the National Eye Institute and conformed to the provisions of the Animal Welfare Act [National Institutes of Health (NIH)/Department of Health and Human Services]. From May to August, squirrels were housed individually on Aspen Sani-Chips bedding (depth, 1 inch) in a holding room set to 72° ± 4°F (22°C) and 30 to 70% humidity. Enrichment consisted of one handful of Enviro-dri bedding and a tunnel made of polyvinyl chloride pipe. Lighting was supplied by full-spectrum bulbs to mimic natural daylight, with a photoperiod matching that prevailing in Oshkosh, WI. From March to August, squirrels were fed cat chow ad libitum. In early July, experimental animals were equipped with subdermal implants for identification and body temperature measurements (BMDS IPTT-300, Bio Medic Data Systems, Seaford, DE).

From September to February, the cat chow ration was regulated to 60 g per week, and their body temperatures were periodically measured. When animals displayed early signs of hibernation/torpor (became sluggish or unresponsive), their cages were transferred to a dark hibernaculum set at 39° ± 3°F (4° to 5°C) and 30 to 70% humidity and monitored daily for short periods under dim red light. Animals that remained torpid for three consecutive days were transferred, with their nest but without food or water, to a 4.4-quart Handi-Box that served as their nest chamber for the duration of their hibernation period. Euthanasia was carried out on animals exposed to 6 liters/min of isoflurane in a 30-liter glass cage lined with wood shavings until deeply sedated, followed by decapitation using a guillotine.

### Method details

#### Retinal sample preparation

Following euthanasia, GS eyes were removed, and posterior segment eye cups were dissected out of enucleated eyes and placed in Hibernate A medium (BrainBits LLC, Springfield, IL). Retinas were dissected out of eye cups, and horizontal sections from dorsal retina that isolated photoreceptor layers were prepared as follows: First, the dorsal region was cut away from the optic nerve head and the inferior retina, and the retinal pigment epithelium was gently removed, taking care to preserve the natural orientation of photoreceptors. The dorsal retina was then cut into small (∼1 to 2 mm^2^) sections, which were then laid flat (photoreceptor side-down) on a standard microscope slide and coated with low–melting point agarose [1% (w/v) in Hepes-buffered Ames’ medium; Sigma-Aldrich, St. Louis, MO] kept at 50°C using a water bath. This was done with the microscope slide placed on an ice pack to speed agarose hardening. Multiple such embedded samples were then placed on a prepared block of standard agarose and coated with more low–melting point agarose to fix them in place. This block was then immersed in the reservoir of a vibratome (Leica VT1000 vibratome, Leica Biosystems, Buffalo Grove, IL) containing room temperature Hibernate A. This block was iteratively sliced and inspected until only photoreceptors remained, at which point a final cut was made to free the photoreceptor-containing slice from the agarose block and invert it photoreceptor side up for imaging.

Agarose-embedded samples were incubated for 30 to 60 min at 4°C with vital dyes diluted in Hibernate A before imaging. Dyes typically included TMRE (50 mM; Thermo Fisher Scientific) and Hoechst 33342 nuclear stain (3 mM; Thermo Fisher Scientific); alternatively, MitoTracker Green (250 nM; Life Technologies) and CellTracker Orange (2 mM; Life Technologies) were used. Following this incubation, samples were gently rinsed with cold Hepes (10 mM)–buffered Ames’ solution (Sigma-Aldrich), placed into fresh Hibernate A with a low concentration of TMRE (5 mM), and returned to the refrigerator until imaging. Incubation at 35°C accelerated dye loading, but samples thrived better at lower temperatures and were thus kept in the refrigerator. For imaging, samples were transferred to dishes containing Hepes-buffered Ames’ and 5 mM TMRE. Ames’ medium was used instead of Hibernate A for imaging because the use of Phenol Red in the latter for pH monitoring may introduce undesired optical effects. For all data presented here, live images were acquired <8 hours following initial euthanasia.

#### Confocal imaging of light transmission through live photoreceptors

Agarose-embedded samples were placed photoreceptor side up in glass-bottom dishes under an upright laser scanning confocal microscope (LSM 510, Carl Zeiss Microscopy, White Plains, NY). Agarose gel has a refractive index ranging from 1.335 to 1.340 (*73*, *74*), similar to the value used in FDTD electromagnetic simulations for the extracellular space surrounding cones (see below).To provide an external transmitted light source during imaging, the condenser was replaced with a custom housing containing a beam combiner to direct light from a collimated “cool white” LED (Mightex LCS-5500-12-22, Mightex Systems, Pleasanton, CA) from below the sample toward the microscope objective, mimicking the normal direction of light passage through the retina. Light from the LED was typically filtered to short (blue) wavelengths using a dichroic color filter (Thorlabs FD1B, 490-nm cutoff, Thorlabs, Newton, NJ); occasionally, this filter was removed to simultaneously observe light concentration by cones across multiple wavelength bands. A glass diffuser was placed between the collimating lens and the blue filter to ensure uniform illumination; preliminary results without a diffuser resulted in the reproduction of the four-emitter LED pattern at the focal length of mitochondria, leading to erroneous measurements of light concentration ability (see also Fig. 5A).

For TMRE-loaded samples, acquisition was performed on two acquisition channels in Zeiss Zen software, with the blue transmitted light filtered through a 435- to 485-nm emission band-pass (“blue channel”), and TMRE was excited using a 561-nm laser and acquired using a 575- to 630-nm band-pass (“TMRE channel”). Transmitted light from the LED was not visible on the TMRE channel or vice versa. 3D volume stacks (“Z-stacks”) were acquired from both channels using standard parameters for TMRE and a pinhole size of 126 mm for the blue channel. Before each acquisition, the intensity of the transmitted LED was adjusted while focusing on the bright puncta of concentrated light above cones to prevent saturation of that channel. Z-stack acquisition times ranged from 10 to 30 min.

Samples including MitoTracker Green were also excited with the 488-nm laser line, and the green channel was acquired behind a 500- to 550-nm band-pass. Blue light from the LED was visible on the green channel but was clearly distinguishable from MitoTracker labeling, which, in turn, was not visible in the transmitted light channel. For acquisitions of light concentration by cones in multiple wavelength bands, no laser excitation was used, but the blue LED filter was removed, and transmitted light was simultaneously acquired on multiple channels filtered to 435 to 485 nm, 500 to 550 nm, and 650 to 710 nm. To spatially register these images with cones, the blue LED filter was then reinserted and the 435- to 485-nm channel reacquired simultaneously with TMRE fluorescence. The two blue channels were then used to later register the two image stacks for analysis.

#### Immunofluorescence

Following live imaging, samples were fixed in 4.0% paraformaldehyde for at least 1 hour and then processed for postfixation imaging to verify cone structures. Samples were blocked with 4% normal donkey serum (Jackson ImmunoResearch Laboratories, West Grove, PA) in phosphate-buffered saline containing 0.1% Triton X-100 (Sigma-Aldrich) for 2 hours. Samples were incubated at least 4 hours with antibodies against the mitochondrial membrane protein Translocase of Outer Mitochondrial Membrane 20 (Tomm20) (ab186734 or ab56783, Abcam, Cambridge, MA), as well as either cone arrestin (Arr3 antibody; sc-54355, Santa Cruz Biotechnology, Dallas, TX) or recoverin (Rcvrn antibody; AB5585, Millipore, Burlington, MA) to label cone photoreceptors. Samples were then incubated at least 4 hours in fluorophore-conjugated secondary antibodies raised in donkey against rabbit, mouse, and/or goat primary antibodies (Jackson ImmunoResearch Laboratories) before mounting. Brief incubation with DAPI (Thermo Fisher Scientific) was included during final sample washes to label nuclei. Low-resolution images taken during live imaging were used to reidentify patches of photoreceptors and verify the absence or presence of cone cell bodies. Images were acquired using an inverted confocal microscope (LSM 780, Zeiss).

#### Serial block-face scanning electron microscopy

Dorsal retinal samples were excised as described above, fixed with 4% paraformaldehyde and 2.5% glutaraldehyde, and provided to Renovo Neural Inc. (Cleveland, OH) for SBEM imaging (*75*). Samples were provided from one active and one hibernating GS as determined above; animals were euthanized in late March when both hibernating and nonhibernating GS were available. The block size and resolution of the resulting SBEM datasets were as follows: active, 28.7 μm by 28.7 μm by 15.0 μm (resolution, 7 nm by 7 nm by 50 nm); hibernating block #1, 21.5 μm by 28.7 μm by 11.0 μm (resolution, 7 nm by 7 nm by 30 nm); and hibernating block #2, 21.5 μm by 28.7 μm by 10.0 μm (resolution, 7 nm by 7 nm by 30 nm).

#### FDTD electromagnetic simulations

The simulation of light transmission through reconstructed cones and their mitochondria was accomplished via the calculation of Maxwell’s equations of electromagnetism (EM) in a discretized grid containing the reconstructed models, into which EM energy oscillating at visible wavelengths was introduced. This process was composed of four fundamental steps: mesh conditioning, model conversion, EM calculations (simulation), and data analysis. Conversion and simulation procedures were performed using custom C++ programs and executed on Biowulf, the NIH supercluster.

##### Preconversion mesh conditioning

Manual segmentation of cone mitochondria produced 3D meshes defining the membrane boundaries of mitochondria and the cell IS. Before conversion, models were rotated to orient the IS orthogonally in the +*Z* direction; for simulations considering the angular dependence of light detection, models were then additionally rotated in the *Y* axis by fixed angles. Mitochondria and cell membrane models containing artifacts that resulted from imaging jitter or segmentation errors were edited in Blender software to remove spurious sharp edges or gaps in meshes. Meshes were further decimated to reduce the number of triangles used in model conversion (see below). In models where the IS membrane was cut off by the edge of the SBEM imaging volume, the cell membrane mesh was edited in Blender to provide a reasonable approximation to the actual expected morphology for simulations. This workflow of converting 3D meshes to FDTD simulation–compatible structures further allowed the construction of de novo alternative mitochondria models for simulation (see fig. S6).

##### Conversion

To translate these oriented meshes into a 3D gridded structure for FDTD simulations (see below), each grid point was tested against the mesh using a point-in-polygon algorithm (*76*), and the resulting grid was stored as a file for later use (fig. S4B). This storage method allowed for the assignment of varying dielectric parameters (refractive indices) to different structures during simulation without the need to recompute the structure. Because the Yee grid formulation used in FDTD simulations computes electric and magnetic fields offset by one-half grid spacing from one another (*38*), this grid discretization was performed at double the resolution specified for actual simulations (i.e., 0.01-μm grid spacing for simulations using a 0.02-μm grid). To speed computation, each mitochondrial mesh was discretized into “slabs” in *Z* containing mesh triangles that intersected a given slab; thus, each point-in-polygon check was simplified to a 2D calculation involving only local triangles. To improve FDTD simulation accuracy using subpixel averaging (*77*), grid points intersecting cell membranes were detected and stored separately with the normal vector of the triangle intersecting that point and the volumetric fill fraction calculated for that point (e.g., for a mitochondrial membrane, the proportion of a given grid point considered “inside” the mitochondrion). These values were later loaded into simulations to initialize the anisotropic dielectric tensor at that point (*77*). Cell and mitochondrial membranes were assumed to have a width of 10 nm; subpixel averaging allowed the elevated refractive indices of membranes to affect light propagation despite their sub–20-nm width.

##### FDTD electromagnetic simulation

Models converted as described above were then preloaded with custom C++ code that used libraries from MEEP 1.2.1 (*38*) as the foundation for FDTD simulations. For simulations, the converted 3D grids were centered in the *X*-*Y* plane with the IS tip placed at a fixed *Z* height of 25 μm; the simulation volume was further extended 10 μm beyond the IS tip to allow space for the calculated EM fields to evolve (Fig. 3 and fig. S4C). If the total converted model grid length was shorter than 25 μm in length, then the first layer of the converted model grid was repeated in the −*Z* direction to provide a continuous homogeneous structure. On all sides, a 1.0-μm-thick perfectly matched layer was established to absorb energy leaving the simulation volume and avoid spurious interference from boundary reflections. Light was launched as a continuous plane wave (Ex-polarized current source with a wavelength in the visible range, typically 450 nm) filling the *X*-*Y* plane and at a *Z* height of either 9 μm (“close source”) or 5 μm (“far source”; see fig. S7). Sources were launched from *Z* = 9 μm unless specified otherwise. The grid was divided into 50 steps per unit length, with models scaled to each length unit equaling 1.0 μm; this corresponds to a grid resolution of 20 nm. To allow EM waves to propagate through the simulation volume, fields were iteratively calculated until 50.0 simulation unit time steps; with a length unit of 1 μm, this corresponds to 50 × 10^−6^ m/*c*_0_ = 1.6678 × 10^−13^ s or 166.78 fs (where *c*_0_ is the speed of light in a vacuum: 299792458.0 m/s).

For these simulations, the dielectric model grid was specified using the relative permittivity ε*_r_* = *n*^2^, where *n* is the refractive index of each material type (e.g., membrane or cytoplasm) in the simulation. Absorption (conductivity of the material) was not modeled. Refractive indices, which may be wavelength dependent, were not modified specifically for the source wavelengths used here. The values for refractive indices were based on previous reports of these values for biological tissues in general (*17*, *18*, *22*, *23*, *78*, *79*) and for photoreceptors in particular (*53*). Here, unless otherwise specified, the refractive indices used were as follows: membrane, 1.46; cytoplasm, 1.36; mitochondria, 1.40; and extracellular space, 1.34. In simulations including procedurally generated cristae structures (see below), the mitochondrial refractive index was instead divided into separate intramembrane and inner matrix compartments with separate refractive indices (see fig. S5).

For simulations including procedurally generated cristae structures (fig. S5), instead of uniform average refractive indices surrounded by a membrane envelope, mitochondria were filled by randomized compartments representing the mitochondrial inner matrix and intramembrane space, separated by a second internal membrane. These cristae were constructed using a sum of 3D cosine functions (*f*) with randomly assigned spatial frequencies and phases, whose values were chosen to qualitatively approximate the spacing of structures present in SBEM images. Grid locations where this sum was positive were assigned as inner matrix; negative values were assigned as intramembrane space. Locations where the absolute value of this sum divided by the magnitude of its gradient was less than half the membrane thickness were assigned as internal membrane (i.e., ∣*f*/ ‖∇*f*‖∣ < 5 nm). Following these assignments, dielectric properties were initialized as described elsewhere in this study. The choices of spatial frequencies were isotropic, i.e., this simplification made no assumption regarding any particular orientation or pattern of cristae structures beyond the intent to create refractive index variations spatially modulated using frequencies on the order of those observed.

##### Analysis

At the end of each simulation, the dielectric ε*_r_* value (see above) and the EM energy density (EnergyDensity) were exported over the entire simulation volume. These files were then converted into image Z-stacks using custom MATLAB scripts; these images were loaded and analyzed as described above for Z-stacks acquired with confocal imaging, where the ε*_r_* stack served as a structural marker analogous to the TMRE channel, and the energy density was measured as for the transmitted (“blue”) channel above. To obtain a measurement of the “background” unfocused light energy, brief simulations were performed with no cone structure (“empty” simulations) using a constant refractive index throughout the simulation volume that was equal to that of the extracellular space.

### Quantification and statistical analyses

#### Cone mitochondria reconstructions and analysis

Images acquired with SBEM were loaded into Reconstruct v1.1 (https://synapseweb.clm.utexas.edu/software-0) (*80*) and/or IMOD v4.9.0 (https://bio3d.colorado.edu/imod/) (*81*) for manual segmentation. Approximately 15 to 20 photoreceptors were present in each sample; only cones with all mitochondria fully visible within the blocks were chosen for reconstruction. Cell membranes were also reconstructed to provide the containing envelope for mitochondria in FDTD simulations. Counts of mitochondria and their volume measurements were obtained using the IMODINFO command line utility included with IMOD and verified with the Print3D plugin for Blender v2.9 (www.blender.org).

To obtain mitochondrial skeletons for alignment analysis, reconstructed models were first loaded into Blender, oriented to point in the +*Z* direction, and rendered as a series of high-contrast images (i.e., a synthetic Z-stack) for further processing. These processed image stacks were then converted into a format readable by KNOSSOS v5.1 (*82*) (www.knossos.app), which was then used to load and annotate each mitochondrion as an individual skeleton. One final skeleton was then annotated using the approximate center point of the cone to define the “primary axis” of each cone cell as a function of *Z*. This two-step approach was adopted instead of directly annotating the SBEM image datasets because of the difficulty of identifying mitochondrial centers in the slicing plane, which was typically oblique to the primary cone axis (see Fig. 2A). Furthermore, renders of reconstructions incorporated the existing “interpretation” of structures that had already been generated from manual contour tracing, thus improving accuracy.

Orientation and alignment analysis were performed on mitochondria skeletons as follows. Skeleton XML data files generated by KNOSSOS were imported using MATLAB R2016b (MathWorks, Natick, MA). Each mitochondrial skeleton was defined by several nodes connected by links (mitochondrial branches). From the bottom (−*Z* direction) to the top (+*Z*) of the cell volume, the *Z* axis was discretized into 0.5-mm steps, and at each of these *Z* steps (i.e., each height layer), we computed the angle between the primary cone branch at that height (see the previous paragraph for the definition of the primary cone skeleton) and each mitochondrial branch passing through that height layer (see Fig. 2D for an illustration of this calculation). For a single mitochondrion passing through this height layer multiple times, each branch was considered separately. Figure S3 depicts the normalized histograms of mitochondria angles as a function of *Z* (height) for each reconstructed cell; the graph in Fig. 2D shows the average histogram across all height levels for mitochondria reconstructions from active versus hibernating GS cones.

#### 3D stack light focusing analysis

Light concentration by cones was measured in Z-stacks using custom routes written in MATLAB R2016b. Because of the similar structure of data between confocal imaging and FDTD electromagnetic simulations (see below), this analysis was applied to both experimental and simulated light transmission data.

Briefly, ellipsoid locations in 3D were annotated by drawing rings around a cone’s TMRE fluorescence in successive images in each stack to delineate the approximate bounds of the mitochondrial bundle. This annotation provided both an estimate of the distal IS tip of the cone and its orientation angle (calculated from an average axis created by the ring centers, weighted by ring area; see fig. S4D). For each such annotated cone, its concentration of light in the transmitted light channel was then annotated by marking both the point of its peak intensity and a center point close to the center of the ellipsoid where the transmitted light forms a “halo” (see Fig. 1D). The line between these two points formed the optical axis along which the light intensity was measured; for each successive image in the stack, the intensity within concentric circles centered upon this axis was calculated. For depth profiles of light intensity such as in Fig. 1D (bottom), the intensity within 0.75 mm of this axis was averaged. To determine the background (unfocused) light level, needed for a calculation of light collection power, an “empty cone” was annotated in each image in a location devoid of structures and featuring a uniform light intensity. The intensity of light collected by annotated cones was then divided by this background intensity to provide a measure of light collection as a function of depth (*Z*) relative to the distal IS tip.

To provide a measure of the degree to which light available to the apparent OSR depends on cone ellipsoid angle (i.e., SCE-like direction sensitivity), a secondary anatomical axis was defined by the mitochondrial ring annotation as described above (see Fig. 5D). The calculation of light intensity in concentric circles centered upon this anatomical axis was repeated as described for the primary optical axis. These two axes allowed a measure of the rotation angle of the cone ellipsoid compared to the optical axis and the calculation of the relative light intensity in the anatomical OSR versus the optical OSR (i.e., the light available to the OS for a cone ellipsoid perfectly aligned to the optical axis; see Fig. 5D). To facilitate comparison to human direction sensitivity parameters, a pupil displacement–to–incident angle conversion of 2.5°/mm was assumed, corresponding approximately to an eye diameter of 25 mm. For simulations of tilted cone reconstructions, angles were measured from the resulting data using this analysis of anatomical versus optical axes instead of assuming the rotation angles used when building and rotating model structures before simulation (see below).

#### Statistics

For comparisons between active and hibernating GS involving reconstructions or simulations of light transmission through reconstructed models, statistical analysis was not performed because of the low effective sample size of *n* = 1 (one retina each from active versus hibernating animals). For comparisons of simulated light gathering by reconstructed cones with varying configurations (e.g., normal versus empty IS), two-sample, two-tailed *t* tests were performed in MATLAB, with significance markings as follows: *P < 0.05, **P < 0.01, and ***P < 0.001. Throughout the present study, focal lengths and peak concentration factors are reported, but statistical comparisons are only presented for OSR concentration, both because the prior two measurements are limited by the distal extent of the data volume (especially so in simulations; see, e.g., Fig. 3C) and because the concentration of light in the OSR is the more physiologically relevant measure for light detection by photoreceptors.

For experimental comparisons of light concentration by cones from horizontally sliced GS retina, the mean difference of light concentration in the apparent OSR between samples from active and hibernating GS was determined by bootstrap statistical sampling with replacement (see fig. S8). Samples were structured to match the original experiment, i.e., four animals each in either the active or hibernating condition, with each animal producing a pool of cones for analysis. The pool sizes for bootstrap resampling were chosen to match those achieved in the real experiments. This sampling was repeated 10,000 times, and the differences between the means of active versus hibernating samples were recorded. The range of differences reported in Fig. 2E corresponds to the 0.5 and 99.5 percentile values over the population of resampled mean differences (i.e., the 99% confidence interval).
