# Supplementary material for: Mitochondria in cone photoreceptors act as microlenses to enhance photon delivery and confer directional sensitivity to light
Source: Sci Adv. 2022 Mar 2;8(9):eabn2070. doi: 10.1126/sciadv.abn2070 (PMC8890704; doi:10.1126/sciadv.abn2070)
Supplement: Supplementary file 1 — Figs. S1 to S8 Table S1 [file sciadv.abn2070_sm.pdf]

Science Advances
AAAS

Supplementary Materials for

Mitochondria in cone photoreceptors act as microlenses to enhance photon delivery and confer directional sensitivity to light

John M. Ball*, Shan Chen, Wei Li*

*Corresponding author. Email: john.ball2@nih.gov (J.M.B.); liwei2@nei.nih.gov (W.L.)

Published 2 March 2022, *Sci. Adv.* **8**, eabn2070 (2022)
DOI: 10.1126/sciadv.abn2070

The PDF file includes:

Figs. S1 to S8
Legend for movie S1
Table S1

Other Supplementary Material for this manuscript includes the following:

Movie S1

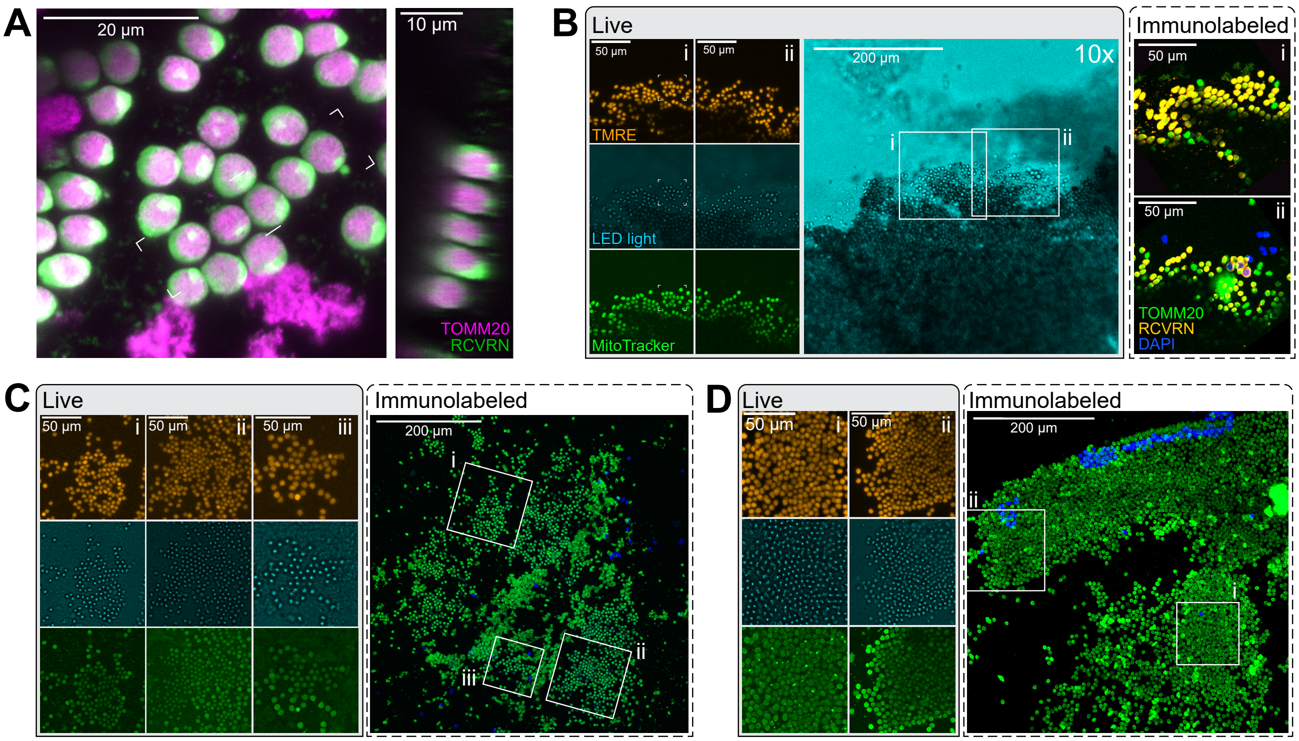

Fig. S1. Verification of ellipsoid isolation in representative samples of horizontally sectioned retinas.

Each group of panels contains images from a sectioned retinal sample during live experiments and in stained images following immunolabeling. Live high-resolution images labeled *i*, *ii*, *iii* are marked in low-resolution images (10x) where available as well as in matching immunolabeled images. Attempts have been made to rotate images to facilitate comparison. Live samples were labeled with TMRE and MitoTracker Green; fixed samples were labeled with TOMM20 (mitochondrial marker), DAPI (nuclei); some samples were also stained for RCVRN (cone marker).

A) Example post-fixation labeled sample highlighting the isolation of mitochondria-bearing ellipsoids. Compare to the cartoons in Fig. 1. Orthogonal projection corresponds approximately to the region enclosed with a dashed box. Note the different color scheme used for this panel compared to the remainder of this figure.

B-D) Example samples labeled for verification. In particular, in panel B, example *i*, the bracketed region corresponds to the cluster of cells highlighted in panel A.

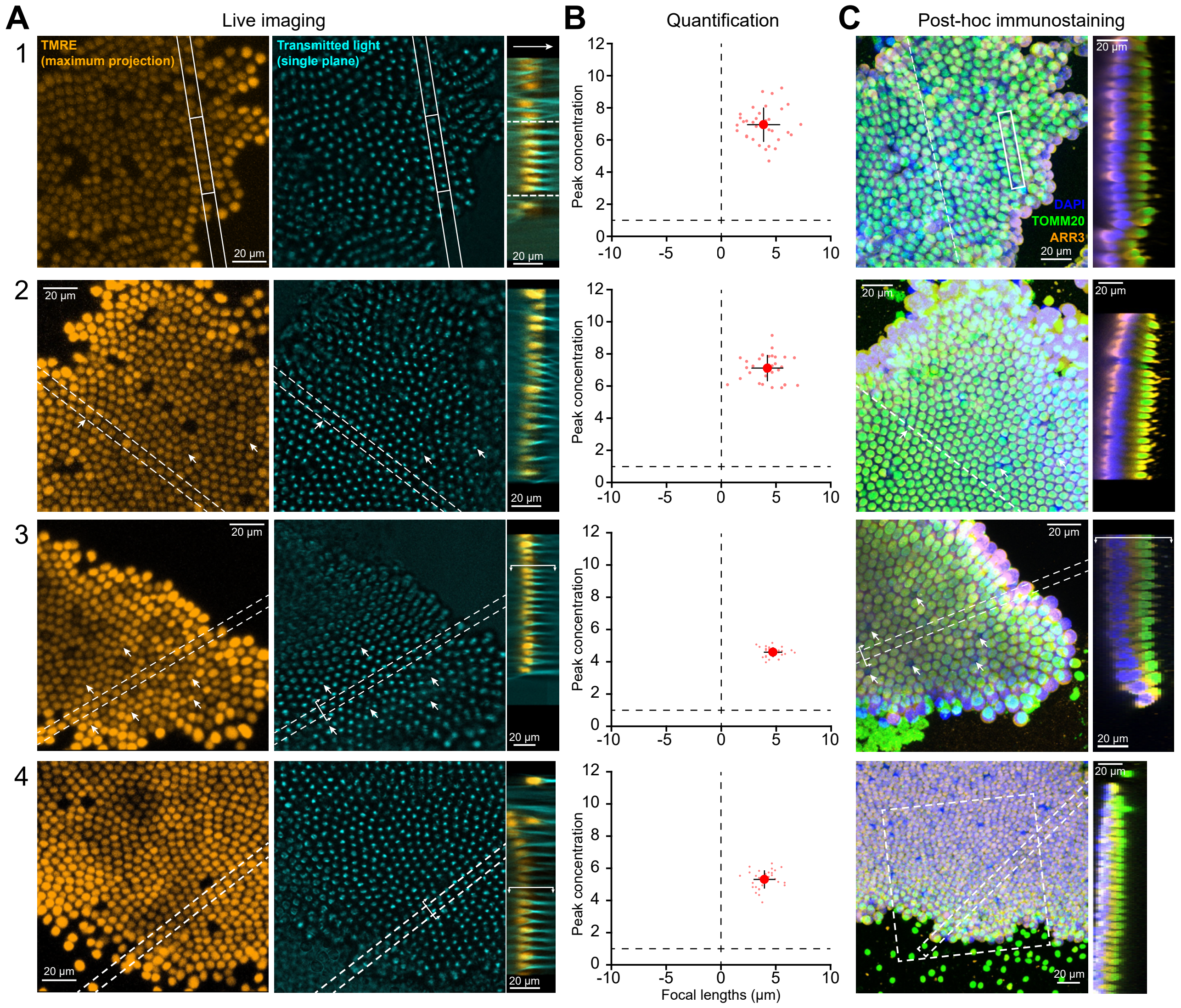

Fig. S2. Light gathering by intact, isolated GS photoreceptors.

Four examples of retinal samples (1-4) where patches of photoreceptors retained intact cell bodies following horizontal cell sectioning. Compare to Fig. S1.

A) Images from live experiments featuring (left-to-right): TMRE maximum projection; single plane of light transmission near focal points of bracketed cones; orthogonal projections of light concentration within the dashed regions. Arrow indicates the direction of light passage through the sample.

B) Quantification of peak light concentration and focal length (distance from putative IS-OS border) for individual cones (small dots) and the average within each sample (large red circle; lines indicate the mean ± standard deviation).

C) Maximum intensity projections of the samples in A immunolabeled following live imaging. These samples possessed clear, intact cell bodies with nuclei. Focusing deeper into the sample during imaging (not shown) revealed a lack of nuclear (DAPI) or mitochondrial (TOMM20) staining, indicating that those inner retinal layers were removed during

vibratome sectioning. Images have been rotated and annotations included to facilitate comparisons among images. Cone arrestin (ARR3) is specific to cone photoreceptors; in particular, in examples *2* and *3*, arrows indicate ARR3-negative rod photoreceptors, which also concentrated light but at focal lengths that differed from their cone neighbors, as the apparent focal points were often not in the same plane. In orthogonal projections from immunolabeled samples, note the inconsistent presence vs. absence of outer segments.

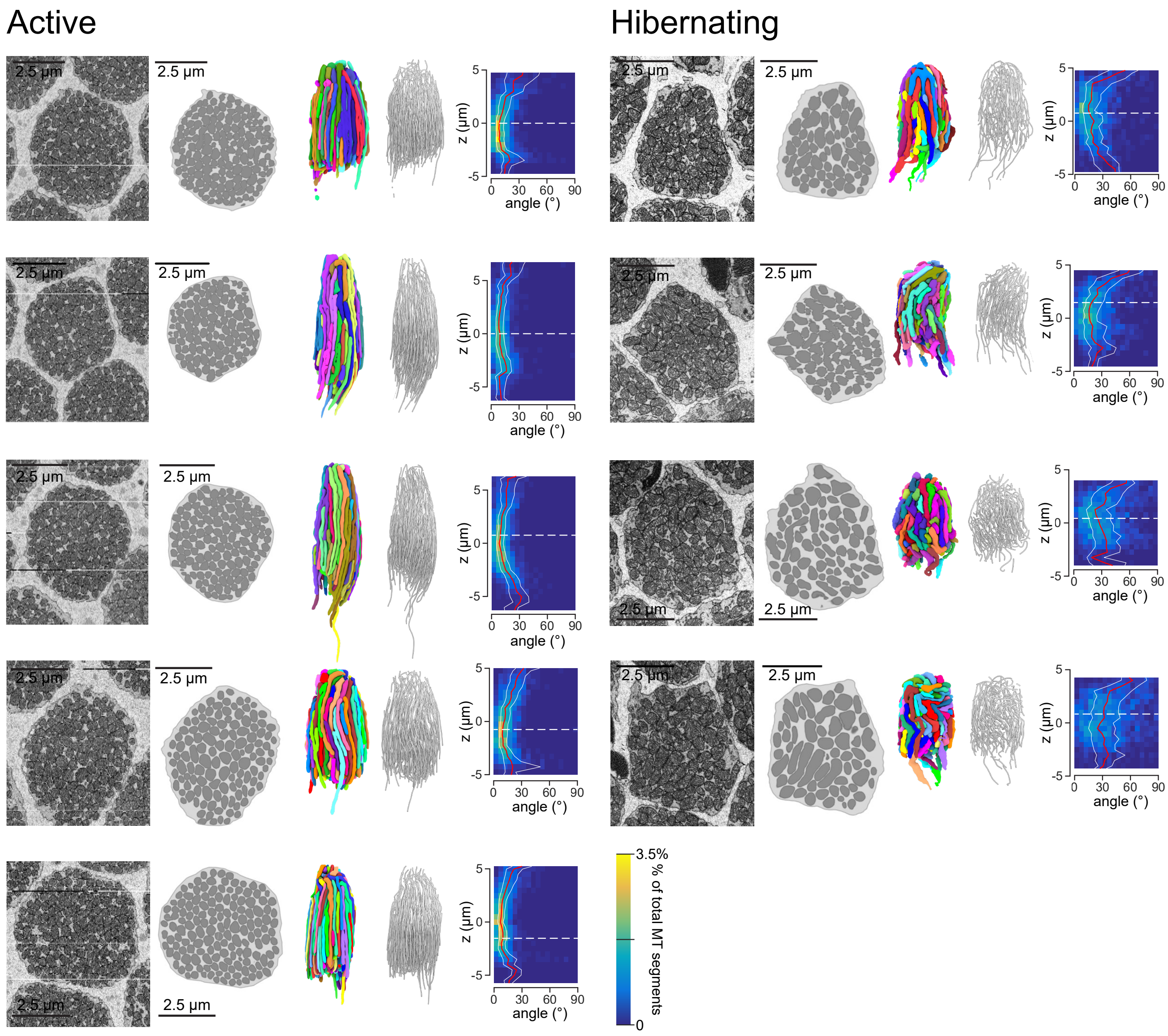

Fig. S3. Alignment analysis of cone mitochondria in reconstructed GS cones.

For each reconstructed cone, the following images or data are presented, from left-to-right: Matching orthogonal cross-sections from original SBEM data and the corresponding dielectric structure from FDTD simulations; 3D model of segmented mitochondria; equivalent skeletonized mitochondrial reconstructions; and heatmaps of mitochondrial alignment analysis. This analysis is described in the Methods, see also Fig. 2D. Here, histograms of mitochondria branch alignment are displayed as a function of cone height (z). Lines indicate the median (red) and lower and upper quartiles (white) for each z value. Color scale encodes the percentage of all mitochondria branches for that cone (scale at bottom of figure applies for all heatmaps). Note that for all cones, mitochondrial orientations are most closely aligned near the center (z ≈ 0); however, this ordered alignment is much more apparent for model cones from active than hibernating GS. Z-axes on alignment heatmaps also serve as scalebars for 3D reconstructions.

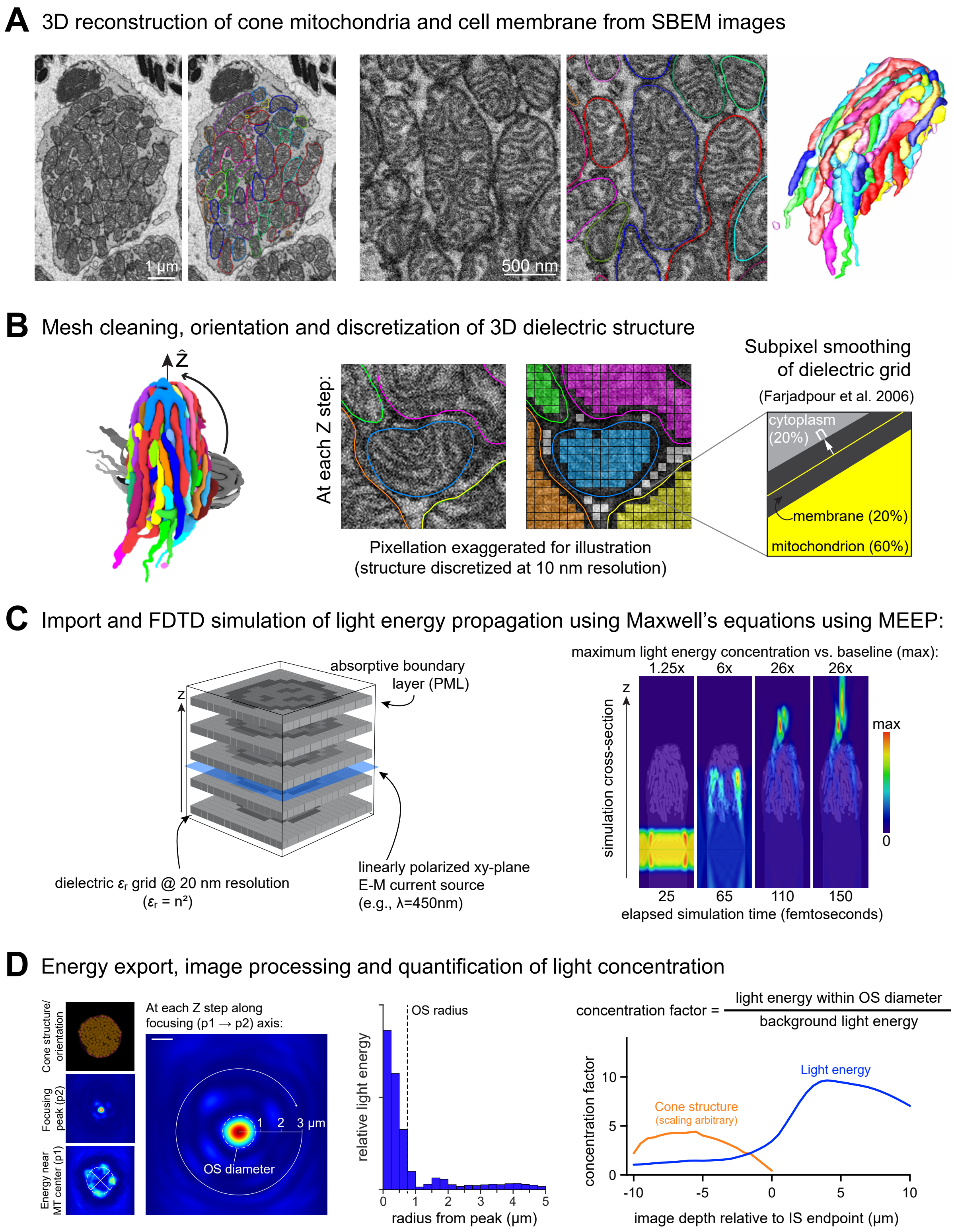

A 3D reconstruction of cone mitochondria and cell membrane from SBEM images
1 µm
500 nm
B Mesh cleaning, orientation and discretization of 3D dielectric structure
Ẑ
At each Z step:
Subpixel smoothing of dielectric grid
(Farjadpour et al. 2006)
cytoplasm (20%)
membrane (20%)
mitochondrion (60%)
Pixellation exaggerated for illustration
(structure discretized at 10 nm resolution)
C Import and FDTD simulation of light energy propagation using Maxwell's equations using MEEP:
absorptive boundary layer (PML)
z
dielectric εr grid @ 20 nm resolution
(εr = n²)
linearly polarized xy-plane
E-M current source
(e.g., λ=450nm)
maximum light energy concentration vs. baseline (max):
1.25x
6x
26x
26x
simulation cross-section
max
0
25
65
110
150
elapsed simulation time (femtoseconds)
D Energy export, image processing and quantification of light concentration
Cone structure/ orientation
Focusing peak (p2)
Energy near MT center (p1)
At each Z step along focusing (p1 → p2) axis:
1
2
3 µm
OS diameter
OS radius
relative light energy
radius from peak (µm)
concentration factor = light energy within OS diameter / background light energy
concentration factor
Light energy
Cone structure
(scaling arbitrary)
image depth relative to IS endpoint (µm)

Fig. S4. Illustration of FDTD electromagnetic simulation procedures.

For further details, see Methods.

A) 3D reconstructions of GS cone mitochondria from SBEM image datasets.

B) Preconditioning of 3D models followed by discretization of the dielectric grid. Grid locations intersecting membranes were encoded with the normal vector of the mesh triangle intersecting that point as well as the volumetric fill fractions inside vs. outside the mesh at that point.

C) Assignment of dielectric relative permittivity assignment in the simulation grid and injection of a linearly polarized current source. For the sample cross-section, note the difference in scaling for each image, which is necessary to show the full range at each time step due to the increasing concentration of light energy over time. The X value for each image indicates the ratio of the maximum energy value in that image to the background light intensity (i.e., 1x indicates no light concentration).

D) Conversion of E-M energy density in the simulation volume to a two-channel Z-stack image and the subsequent light concentration analysis.

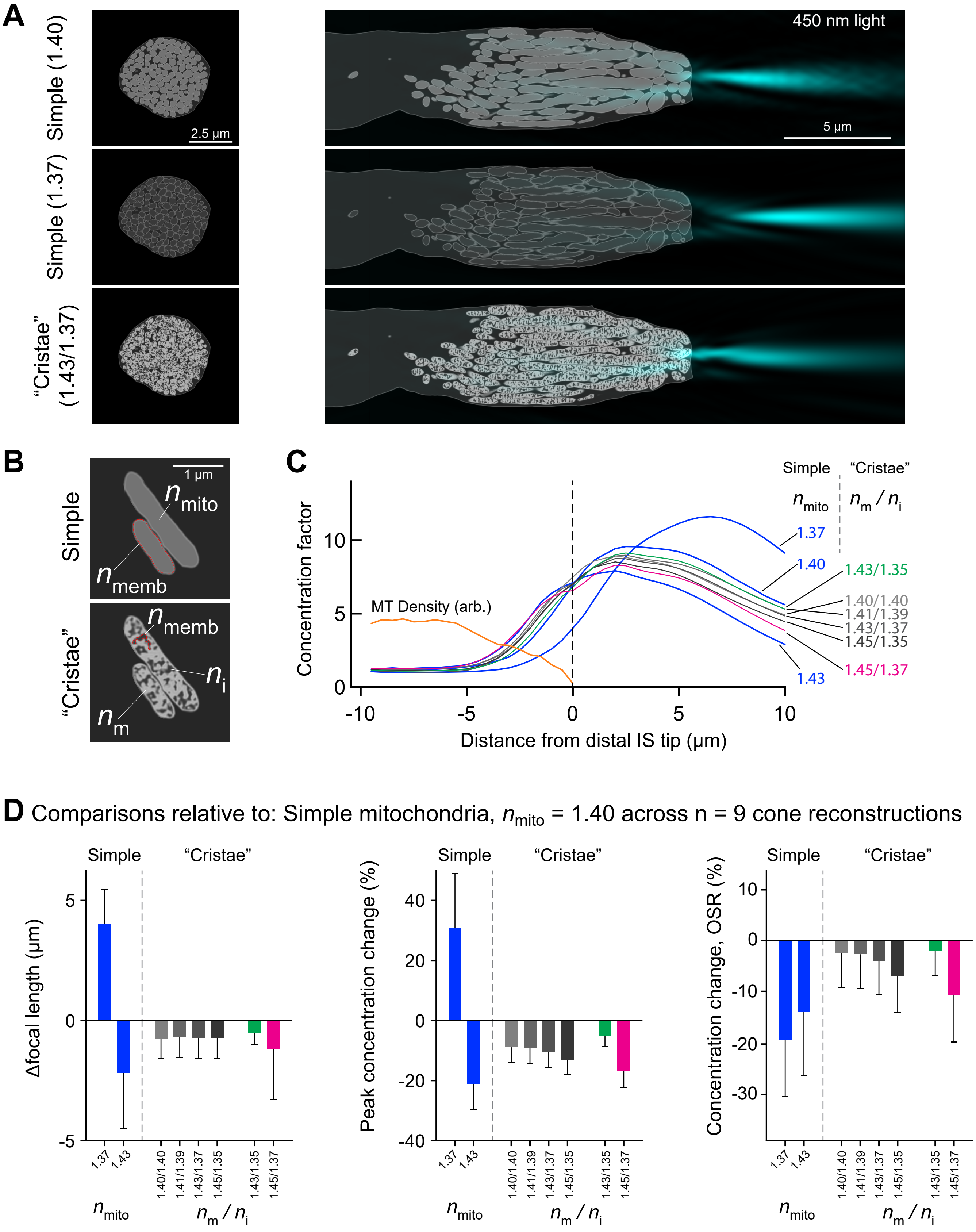
A
Simple (1.40)
Simple (1.37)
"Cristae" (1.43/1.37)
2.5 µm
450 nm light
5 µm
B
Simple
"Cristae"
1 µm
n_{mito}
n_{memb}
n_i
n_m
C
Simple
"Cristae"
n_{mito}
n_m / n_i
Concentration factor
MT Density (arb.)
Distance from distal IS tip (µm)
1.37
1.40
1.43
1.43/1.35
1.40/1.40
1.41/1.39
1.43/1.37
1.45/1.35
1.45/1.37
D Comparisons relative to: Simple mitochondria, n_{mito} = 1.40 across n = 9 cone reconstructions
Δfocal length (µm)
Peak concentration change (%)
Concentration change, OSR (%)
Simple
"Cristae"
1.37
1.43
1.40/1.40
1.41/1.39
1.43/1.37
1.45/1.35
1.43/1.35
1.45/1.37
n_{mito}
n_m / n_i

Fig. S5. Dependence of simulated light focusing upon mitochondrial refractive properties.

Simulations in this figure were performed in one of two modes: “Simple” or “Cristae”. “Simple” simulations were as described elsewhere in the present study; mitochondria were modeled with uniform average refractive indices (n_{mito}) separated from the cytosol by a membrane envelope with refractive index n_{memb}. “Cristae” simulations were modified by dividing mitochondria into separate inner matrix and inter-membrane compartments with refractive indices n_{m} and n_{i}, respectively, separated by a second internal membrane (n_{memb} = 1.46, as elsewhere in the present study). For Simple simulations, the average n_{mito} is indicated, whereas for Cristae simulations, refractive indices are instead indicated as $n_{\mathrm{m}}/n_{\mathrm{i}}$.

A) Images from examples depicting top-down (horizontal) and vertical cross-sections of simulations of an active GS cone under three different refractive configurations; note that higher refractive indices are encoded by brighter colors.

B) Example comparison of internal mitochondrial structures in Simple vs. Cristae simulations.

C) Light concentration profiles for various refractive index configurations using the example cone shown in this figure. Simple (n_{mito} = 1.40) is the configuration for simulations used elsewhere in this study. For Simple simulations (blue), increased n_{mito} shortens the focal length but decreases peak light concentration; conversely, lower n_{mito} increases light concentration, but at longer focal lengths. For Cristae simulations, the addition of the inner membrane without otherwise modifying the average mitochondrial refractive index (i.e., n_{m} = n_{i} = 1.40) decreased light concentration by a small amount. From there, balanced changes to the refractive indices of the two compartments (i.e., increases in n_{m} with matching decreases in $n_{\mathrm{i;}}$ indicated by gray lines) produced only modest effects, even with n changes of ± 0.05 (profiles in grayscale colors). In contrast, unbalanced refractive index adjustments (green, magenta) had effects equivalent to increasing or decreasing the average refractive index of mitochondria; e.g., n_{m} = 1.43 with n_{i} = 1.35 produced focusing changes similar to a that of a slight decrease in overall refractive index.

D) Bar graphs indicating the average changes in light-gathering measures for Simple and Cristae simulations across all cone reconstructions (n = 9 models) in comparison to the canonical Simple (n_{mito} = 1.40) configuration. Interestingly, note that OSR light intensity in Simple simulations was decreased both by an increase or a decrease in n_{mito} due to the cumulative trade-off between peak concentration and focal length.

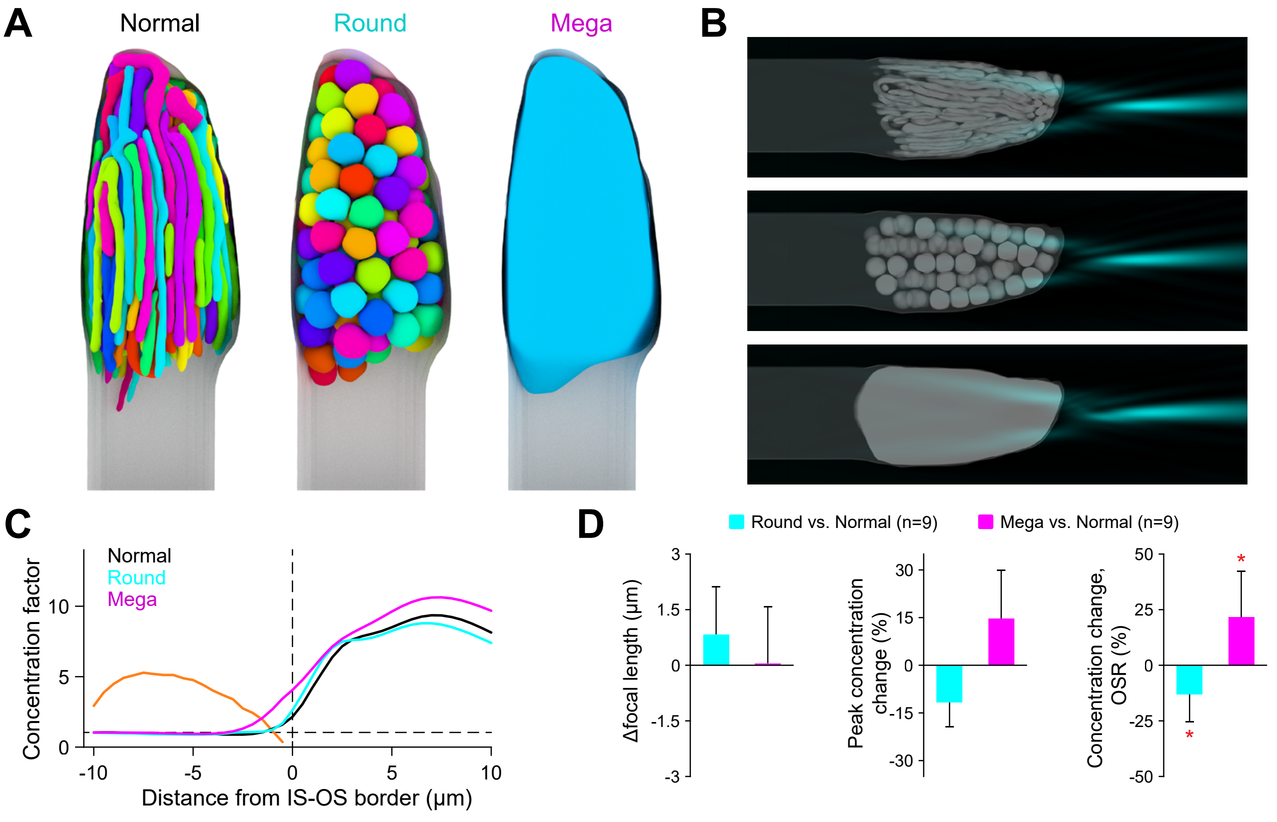

Fig. S6. Simulated light concentration by alternate cone mitochondria configurations.

A) 3D renders of alternate mitochondria configurations for an example cone from active GS.

B) Simulation cross-sections of light focusing by the cone models in *A*.

C) Light concentration factor profile corresponding to the simulations in *B*. Orange curve to the left of the dashed axes indicates mitochondrial location in the simulation volume, mimicking the TMRE signal shown in live imaging.

D) Bar graphs depicting the change in light-gathering measures for “round” or “mega” simulations compared to the “normal” configuration across all cone models (n = 9). Note that light concentration by “round” mitochondria was inferior to the normal configuration, whereas the “megamitochondrion” configuration was optically superior. OSR concentration factor statistics are the result of one-sample t-tests computed for all cone models in each condition (Round or Mega mitochondria) to a mean of zero (i.e., an average zero change in OSR concentration factor).

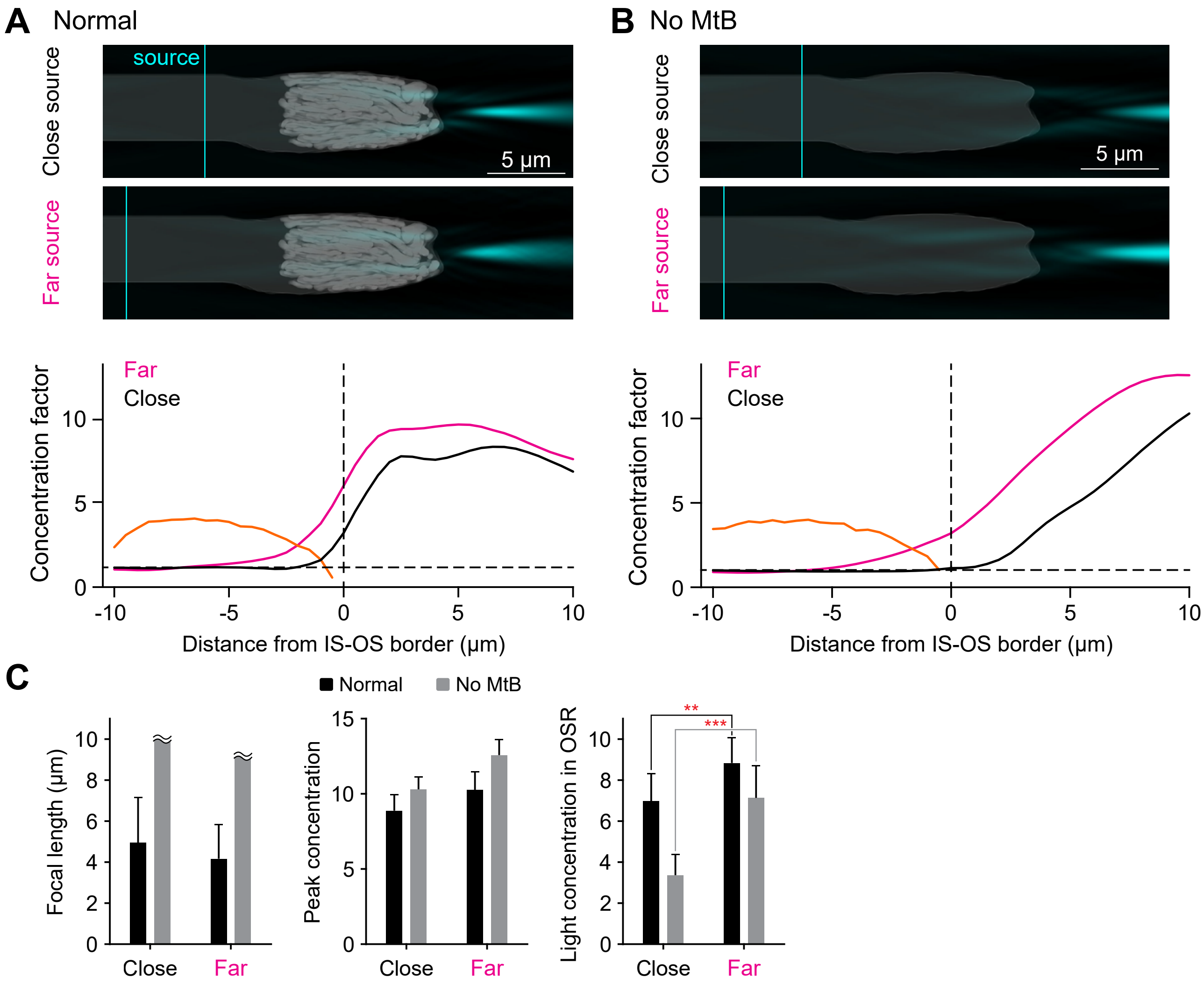

Fig. S7. Normalization of focal lengths by cone mitochondria for varying inner segment length.

A) Example cross-sections and light concentration factor profiles from normal simulations in which the E-M current source was retracted 4 mm further away from cone mitochondria ("far" source), thus mimicking the optical consequences of lengthening the cone inner segment. Simulations were performed as described elsewhere in this study. Orange curves to the left of the dashed axes indicate mitochondrial location in the simulation volume, mimicking the TMRE signal shown in live imaging.

B) Simulations as in *A* that instead featured empty cones (No MT).

C) Comparison of changes in light-gathering measures for normal simulations and those without MT considering close vs. far light sources. Note that IS lengthening shortened focal lengths and increased OSR light intensity (close vs. far), but the presence of mitochondria decreased the focusing difference between these effective inner segment lengths (Normal vs. No MT).

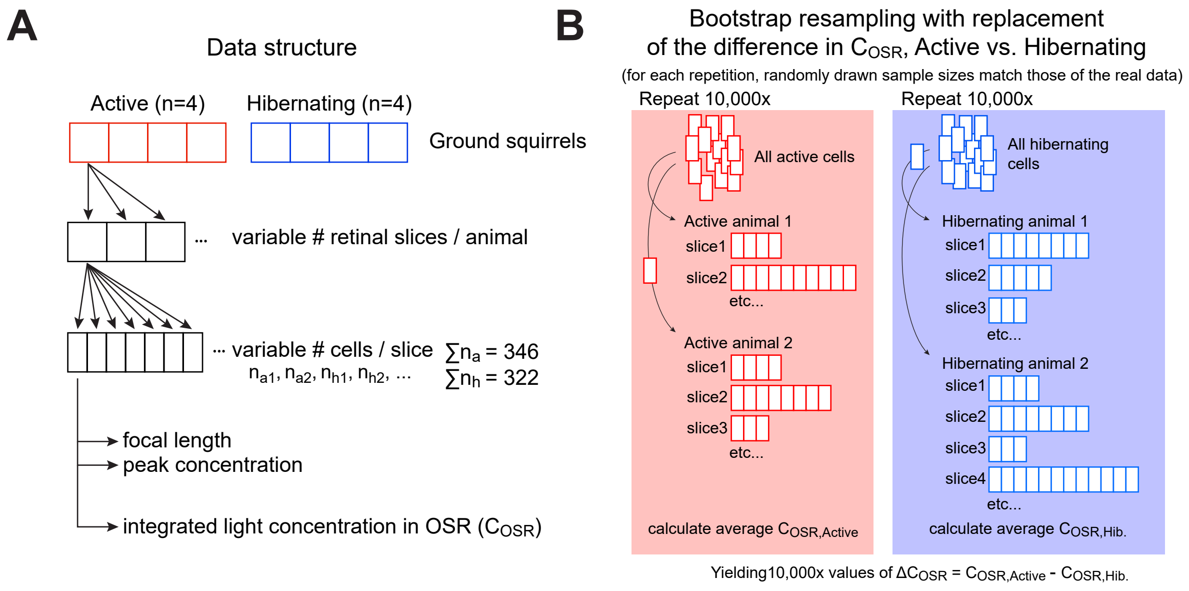

Fig. S8. Data structure and bootstrap statistics for experimental light concentration by cones.

A) Structure of sampled imaging data for cone photoreceptor light concentration in active and hibernating GS. n_{a1} represents the number of photoreceptors analyzed for active GS slice 1, etc.

B) Bootstrap resampling scheme for statistical analysis of OSR light concentration differences between active and hibernating GS cone photoreceptors. The 99% confidence interval for ΔC_{OSR} is reported as the 0.5% and 99.5% percentile of the 10,000 values thus generated.

Movie S1. Animation depicting light focusing by GS cones.

TMRE signal shown in orange, blue LED light in cyan. The whole-mount view on the left is a single focal plane from the z-stack chosen near the peak intensity of light focusing for the majority of cones in the field of view. The red line indicates the vertical plane from the z-stack being shown in the orthogonal projection on the right.

Table S1. Table of reagents, equipment, and software used.

Reagents	Vendor	Cat #	Notes
Hibernate A medium	BrainBits LLC	N/A	Supplemented with Phenol Red
Ames' medium	Sigma	A1420	Supplemented with 10 mM HEPES buffer
HEPES 1M	ThermoFisher	15630-130	
Low gelling temperature agarose	Sigma	A0701-25G	1% w/v in HEPES-buffered Ames' medium
Hoechst 33442	ThermoFisher	H3570	1:5000 from 16 mM stock (water)
Tetramethylrhodamine, Ethyl Ester	ThermoFisher	T669	1:1000 from 50 mM stock (DMSO)
MitoTracker Green	ThermoFisher	M7514	1:4000 from 1 mM stock (DMSO)
CellTracker Orange	ThermoFisher	C34551	1:1000 from 2 mM stock (DMSO)
Tomm20 antibody (mouse monoclonal)	abcam	ab56783	1:500 dilution
Tomm20 antibody (rabbit monoclonal)	abcam	ab186734	1:500
Cone arrestin (Arr3) antibody	Santa Cruz Biotech	sc-54355	1:500
Recoverin antibody	Millipore	AB5585	1:500
DAPI	ThermoFisher	D3571	1:1000
Normal donkey serum	Jackson Immunology Research	017-000-121	4% in phosphate buffer (PB)
Triton X-100	Sigma	T8787	0.1% in PB
**Equipment**	**Manufacturer**	**Model #**	**Notes**
LSM 510	Zeiss	LSM 510	Live imaging
LSM 780	Zeiss	LSM 780	Structure verification
Leica Vibratome	Leica	VT1000	
Mightex "cool white" collimated LED	Mightex	LCS-5500-12-22	
Blue Thorlabs filter	ThorLabs	FD1B	~490nm cutoff
**Software**	**Author**	**Version**	**Notes**
MATLAB R2016b	Mathworks	R2016b	Data analysis, model conversion
Zen (LSM 510)	Zeiss		Image acquisition
Zen (LSM 780)	Zeiss		Image acquisition
MEEP https://meep.readthedocs.io/en/latest/	(*38*)	v1.2.1	C++ libraries used in custom code
Reconstruct https://synapseweb.clm.utexas.edu/software-0	(*80*)	v1.1	Reconstructions
IMOD (https://bio3d.colorado.edu/imod)	(*81*)	v4.9.0	Reconstructions, structure analysis
Blender (https://www.blender.org/)	Blender Foundation	v2.79	Model editing, rendering, structure analysis
KNOSSOS (https://knossos.app/)	(*82*)	v5.1	Skeletonization
